# Supplementary material for: Persistent Organic Pollutant Levels in Maternal and Cord Blood Plasma and Breast Milk: Results from the Rio Birth Cohort Pilot Study of Environmental Exposure and Childhood Development (PIPA Study)
Source: Int J Environ Res Public Health. 2022 Dec 31;20(1):778. doi: 10.3390/ijerph20010778 (PMC9819236; doi:10.3390/ijerph20010778)
Supplement: Supplementary file 1 [file ijerph-20-00778-s001.zip › ijerph-2050766-supplementary.pdf]

Persistent organic pollutant levels in maternal, cord blood plasma and breast milk: Results from the Rio Birth Cohort Pilot Study of Environmental Exposure and Childhood Development (PIPA Study)

Supplemental Material

Table S1. Calibration parameters and limits for OCs and PCBs.

Organochlorine and PCB	Slope	Linear Coefficient	Linearity	Recovery (%)	LOQ ng mL-1	LOD ng mL-1
2,4'-DDD	164424	-105963	0.9847	102	0.189	0.063
2,4'-DDE	133466	-104848	0.9824	102	0.201	0.066
2,4'-DDT	206667	-144347	0.9658	105	0.063	0.021
4,4'-DDD	212082	6675	0.936	101	0.09	0.03
4,4'-DDE	111655	-33095	0.9736	99	0.205	0.068
4,4'-DDT	205722	-229041	0.9562	102	0.145	0.048
Aldrin	4576	-8718	0.9854	99	0.818	0.27
alfa-Chlordane	11662	-14012	0.9787	97	0.715	0.236
alfa-Endosulfan	5767	-9126	0.9631	105	1.125	0.371
alfa-HCH	92536	-70174	0.9832	94	0.201	0.066
beta-Endosulfan	5994	-7198	0.9946	101	0.619	0.204
beta-HCH	58035	-39473	0.9865	97	0.053	0.017
delta-HCH	30644	-25637	0.9838	97	0.172	0.057
Dicofol	89022	-93845	0.9921	94	0.101	0.033
Dieldrin	7141	-14131	0.988	103	0.476	0.157
Endosulfan sulfate	29623	-31105	0.962	102	1.009	0.333
Endrin	7571	-13910	0.9906	96	0.734	0.242
gama-Chlordane	10049	-16507	0.9634	93	1.419	0.468
gama-HCH	39580	-27352	0.9833	96	0.212	0.07
Heptachlor	32598	-27788	0.9783	96	0.162	0.053
Heptachlor epoxide	8442	-14194	0.9808	99	1.189	0.392
Hexachlorobenzene	278528	-48803	0.983	94	0.045	0.015
Metoxichlor	99450	-96189	0.9648	99	0.159	0.052
Mirex	74864	-68552	0.9764	101	0.149	0.049
Pentachloroanisole	96295	-92860	0.9726	95	0.143	0.047
trans-Nonachlor	5848	-7051	0.9825	103	0.64	0.21
PCB 28	274097	-134898	0.9774	95	0.19	0.06
PCB 31	484003	-637790	0.9656	94	0.17	0.06
PCB 52	124862	-37259	0.9731	95	0.19	0.06
PCB 77	68743	-178717	0.9412	99	0.25	0.08
PCB 101	35008	-95681	0.9525	96	0.25	0.08
PCB 105	44770	-78194	0.9452	100	0.24	0.08
PCB 118	52787	-83860	0.9652	98	0.19	0.06

PCB 126	33000	-72587	0.9587	87	0.19	0.06
PCB 128	10601	-18607	0.9674	99	0.27	0.09
PCB 138	24033	-70547	0.9461	97	0.3	0.1
PCB 153	42680	-36395	0.9583	109	0.05	0.02
PCB 156	6723	-40544	0.9481	107	0.17	0.06
PCB 169	6078	-348809	0.9552	99	1.08	0.36
PCB 170	9318	-27753	0.9458	95	0.96	0.32
PCB 180	65180	-34198	0.9886	94	0.2	0.07

Table S2. Estimated HQ from breast milk described in the literature.

Country	Estimated HQ	Situation	Reference
Brazil	Mean[a]<br>0.28[b]<br>0.14[c]<br>Highest value[a]<br>104.85[b]<br>52.43[c]	Riverine mothers	[22]
Brazil	$3.14x10^{-4}$	Mothers from urban area and countryside	[23]
Ethiopia	Mean[a]<br>58.80[b]<br>29.40 [c]	Mothers from subsistence farming areas	[21]
India	Mean[a]<br>21.44[b]<br>10.72[c]	Mothers from rural and urban areas	[54]
Poland	Primipara mothers<br>Mean<br>0.30[b]<br>0.15[c]<br>Highest Value<br>2.5[b]<br>1.23[c]<br>Multipara mothers<br>Mean<br>0.34[b]<br>0.17[c]<br>Highest Value<br>3.20[b]<br>1.60[c]	Primipara and multipara mothers	[55]
Turkey	Van<br>Mean<br>25.32[b]<br>12.66[c]<br>Manisa<br>Mean<br>24.08[b]<br>12.04[c]	Mothers living in Van and Manisa	[56]
USA	28[b]<br>14[c]	Mothers from Pitt, Durham, and Wake, North Carolina	[57]
New Zealand	North region<br>12.32<br>South region<br>6.16	Mothers from Auckland and Christchurch	[58]

[a] Adjusted by 4% of lipid; [b] IRIS EPA estimation; [c] FAO/WHO estimation
